# Public health impact and cost-effectiveness of intranasal live attenuated influenza vaccination of children in Germany

**DOI:** 10.1007/s10198-014-0586-4

**Published:** 2014-05-25

**Authors:** Oliver Damm, Martin Eichner, Markus Andreas Rose, Markus Knuf, Peter Wutzler, Johannes Günter Liese, Hagen Krüger, Wolfgang Greiner

**Affiliations:** 1 Department of Health Economics and Health Care Management, Bielefeld School of Public Health, Bielefeld University, Universitätsstraße 25, 33615 Bielefeld, Germany; 2 Department of Medical Biometry, University of Tübingen, Tübingen, Germany; 3 Epimos GmbH & Co. KG, Dusslingen, Germany; 4 Department of Pulmonology, Allergy and Infectious Diseases, Children’s Hospital, Goethe University, Frankfurt, Germany; 5 Department of Children and Adolescents, Dr. Horst Schmidt Klinik, Wiesbaden, Germany; 6 Paediatric Infectious Diseases, University Medicine Mainz, Mainz, Germany; 7 Institute of Virology and Antiviral Therapy, University Hospital, Friedrich Schiller University of Jena, Jena, Germany; 8 Department of Paediatric Infectious Diseases, University Children’s Hospital Würzburg, Würzburg, Germany; 9 Medical Department, AstraZeneca GmbH, Wedel, Germany

**Keywords:** Influenza, Vaccination, Children, Cost-effectiveness, Dynamic transmission model, Germany, I18

## Abstract

In 2011, intranasally administered live attenuated influenza vaccine (LAIV) was approved in the EU for prophylaxis of seasonal influenza in 2–17-year-old children. Our objective was to estimate the potential epidemiological impact and cost-effectiveness of an LAIV-based extension of the influenza vaccination programme to healthy children in Germany. An age-structured dynamic model of influenza transmission was developed and combined with a decision-tree to evaluate different vaccination strategies in the German health care system. Model inputs were based on published literature or were derived by expert consulting using the Delphi technique. Unit costs were drawn from German sources. Under base-case assumptions, annual routine vaccination of children aged 2–17 years with LAIV assuming an uptake of 50 % would prevent, across all ages, 16 million cases of symptomatic influenza, over 600,000 cases of acute otitis media, nearly 130,000 cases of community-acquired pneumonia, nearly 1.7 million prescriptions of antibiotics and over 165,000 hospitalisations over 10 years. The discounted incremental cost-effectiveness ratio was €1,228 per quality-adjusted life year gained from a broad third-party payer perspective (including reimbursed direct costs and specific transfer payments), when compared with the current strategy of vaccinating primarily risk groups with the conventional trivalent inactivated vaccine. Inclusion of patient co-payments and indirect costs in terms of productivity losses resulted in discounted 10-year cost savings of €3.4 billion. In conclusion, adopting universal influenza immunisation of healthy children and adolescents would lead to a substantial reduction in influenza-associated disease at a reasonable cost to the German statutory health insurance system. On the basis of the epidemiological and health economic simulation results, a recommendation of introducing annual routine influenza vaccination of children 2–17 years of age might be taken into consideration.

## Background

Annual seasonal influenza epidemics are associated with considerable health and economic consequences worldwide [1–3]. Several studies have particularly underlined the clinical and socioeconomic impact of influenza in children [4–10]. A recently published review concluded that influenza-related mortality in children is limited, but influenza-associated paediatric hospitalisation rates are high and parental work loss is substantial [11].

Influenza is usually a self-limiting condition with systematic and respiratory symptoms that last up to 7 days in most people. However, influenza infection can also result in moderate to severe complications, such as acute otitis media (AOM), bronchitis, pneumonia and other respiratory diseases potentially leading to hospitalisation. In rare cases, influenza can lead to severe non-pulmonary complications, e.g. cardiac and neurologic complications [12]. Several management strategies including vaccination and antiviral treatment are available to cope with seasonal influenza epidemics. Vaccination is the most effective option for preventing influenza and related illnesses [13].

Currently, there are two types of seasonal influenza vaccines licensed for use in Europe: injectable trivalent inactivated influenza vaccine (TIV) and the nasal spray live attenuated influenza vaccine (LAIV). While LAIV is indicated for children and adolescents 2–17 years of age, non-adjuvanted TIV is licensed for individuals aged 6 months or over. According to two recently published meta-analyses [14, 15] LAIV showed high levels of protection against culture-confirmed influenza in children. Furthermore, LAIV efficacy in children was consistently found to be higher than efficacy estimates for TIV [14]. In addition, LAIV was associated with a more sustained duration of protection than TIV [16–18]. Moreover, a survey of preferences for influenza vaccine attributes including efficacy and mode of administration among children aged 8–12 years found that 79 % of children favoured the LAIV-like vaccine profile over the TIV characteristics [19]. As part of the proposed revisions to the 2005 World Health Organization (WHO) position paper on influenza vaccines, the Strategic Advisory Group of Experts (SAGE) Influenza Working Group [20] recently recommended use of LAIV instead of TIV for children aged 2–5 years because of enhanced levels of protection in this age group.

In Germany, annual influenza vaccination is mainly recommended from the age of 60 years and for people with underlying chronic conditions [21]. Similar influenza immunisation policies have been adopted in all other EU member states. Up to now, only a few countries actually recommend vaccinating healthy children against seasonal influenza. However, the number of countries introducing universal influenza vaccination of children is growing [22, 23]. Current childhood influenza immunisation recommendations use different age ranges for defining the target group. For example, Canada adopted universal vaccination for all children aged 6–23 months of age, whereas in the USA influenza vaccination is recommended for children and adolescents from 6 months to 18 years of age. Within Europe, merely nine countries (Austria, Estonia, Finland, Latvia, Malta, Poland, Romania, Slovakia and Slovenia) have already established programmes for vaccinating healthy children against influenza, targeting children of different age groups from 6 months to 2 years up to 6 months to 18 years [23, 24]. In Germany, only the state of Saxony recommends vaccination of all children older than 6 months of age against influenza [25]. The UK’s Joint Committee on Vaccination and Immunisation (JCVI) recently recommended annual vaccination of children aged 2 to under 17 years against seasonal influenza. Furthermore, the JCVI pointed out that LAIV should be the vaccine of choice when implementing the extension of the annual influenza vaccination programme to healthy children [26].

In recent years, there has been increasing interest in modelling both the spread of influenza infection and the economic assessment of potential management strategies. However, to the best of our knowledge, no previous study has investigated the epidemiological and health economic impact of a general influenza vaccination programme among children with LAIV in the German health care setting. Thus, the objective of the present modelling study was to compare the epidemiological and health economic consequences of an additional LAIV-based routine influenza vaccination programme in children (2–17 years) with the current practice of primarily vaccinating high-risk groups with TIV. We applied a dynamic transmission framework because current evidence suggests that routine childhood vaccination against influenza could provide indirect benefits to the community [27–32]. For instance, a recent US database analysis of hospitalisation records of older adults and influenza vaccination coverage in children and older adults revealed that vaccination of young children against influenza was associated with a reduction in the influenza- and pneumonia-associated burden of disease in the older population [33].

## Materials and methods

### Model features

We used Microsoft Excel and Java to develop a mathematical model which simulates the transmission of seasonal influenza as well as different courses of disease and evaluates the cost-effectiveness of different vaccination strategies. The economic analysis takes three perspectives: (1) a societal perspective (including all direct and indirect costs), (2) a narrow third-party payer perspective (including reimbursed direct health care costs only) and (3) a broad third-party payer perspective (accounting for all reimbursed direct costs and specific transfer payments). In the German health care system the third-party payer is represented by statutory health insurance funds. Cost-effectiveness results are expressed in terms of incremental cost-effectiveness ratios (ICERs) and return rates per euro invested. Cost-effectiveness ratios were calculated as the incremental cost per additional quality-adjusted life year (QALY). The approach of using return rates per euro invested is also known as the concept of benefit–cost ratio (BCR) which represents the ratio of monetary benefits over incremental intervention costs and is obtained by dividing the estimated net savings by the estimated net costs of the intervention. In our analyses, this ratio is equal to the costs of influenza infections averted by the childhood immunisation programme divided by the programme costs. The costs associated with the childhood vaccination programme include those of the vaccine as well as administration costs and costs for treating adverse events. Return rates over 1.0 indicate that the childhood vaccination programme yields overall cost savings to the health care system or the society. Details of the modelling approach are as follows.

### Model design

We used a dynamic modelling approach to simulate the transmission of influenza and to estimate the impact of several influenza vaccination strategies on a population level in Germany [34]. This deterministic and age-structured compartmental model adapting an extended SEIRS (susceptible–exposed–infectious–recovered–susceptible)-type disease transmission model is based on over 4,000 ordinary differential equations. It distinguishes between influenza A and influenza B infections and roughly divides the underlying population into seven distinct groups: individuals who are maternally protected (M), susceptible (S), exposed in terms of being infected, but not yet infectious (E), infectious (I), recovered and immune (R) and subjects who are immune after LAIV immunisation (V_LAIV_) or immune following TIV immunisation (V_TIV_).

**Fig. 1:**
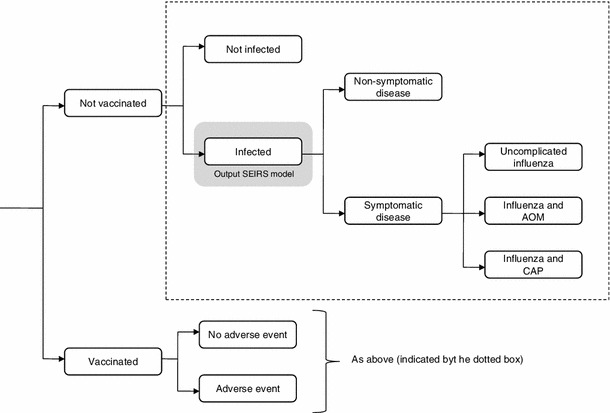
Model structure of the influenza outcome subtree. *AOM* acute otitis media, *CAP* community-acquired pneumonia

In addition, we constructed an influenza outcome subtree to account for various respiratory disease patterns and related health care consumption. The model pathways include both episodes of uncomplicated but symptomatic influenza and more complicated courses of disease. Regarding potential respiratory complications, the model takes account of AOM and of community-acquired pneumonia (CAP). Children who are vaccinated have chances of developing local or systemic adverse events. For adult vaccinees, no side effects requiring treatment were considered. A simplified diagram of the model structure is provided in Fig. 1.

### Population

The simulated population is based on current demographic data reported by the Federal Statistical Office of Germany [35]. The results of our population forecast are similar to the official results of the 12th coordinated population projection for Germany excluding migration [36]. In the economic part of the model, the population was divided into six age and risk groups: children under 2 years of age (CH 1), children 2–6 years of age (CH 2), children 7–12 years of age (CH 3), adolescents 13–17 years of age (CH 4), otherwise healthy adults 18–59 years of age (OHA) and at-risk patients (ARP) comprising individuals with underlying chronic conditions 18–59 years of age and the elderly population aged 60 years and over. The proportion of individuals with a chronic condition (such as chronic respiratory disease, circulatory disorders, endocrine disorders, chronic liver and renal disorders, malignant neoplasms) under the age of 60 was estimated to be 7.6 and 16.6 % for adults 18–44 years of age and adults 45–59 years of age, respectively [37]. Childhood age groups were not differentiated by risk status, because risk-stratified vaccination coverage estimates were not available for children.

### Key parameters of the transmission model

We employed the German mixing matrix of the POLYMOD study (Improving Public Health Policy in Europe Through Modelling and Economic Evaluation of Interventions for the Control of Infectious Diseases) to consider age-specific contact patterns in our simulations [38]. For the base-case analysis, we used a seasonally varying basic reproduction number with an average of 1.6 which lies well in the range of values (0.9–2.1) estimated by Chowell et al. [39] regarding seasonal influenza epidemics across three countries. According to a review of studies describing the timelines of influenza virus infection [40], we assumed a 1-day period of latent infection (prior to becoming infectious) followed by a 5-day period of viral shedding. We further assumed that 30 % of newborns were protected by maternal antibodies, applying an average duration of 4 months for maternal protection. Following the approach of Vynnycky et al. [41], the average duration of acquired immunity after natural infection (modelled by a constant rate of loss of protection per year) was assumed to be 6 and 12 years for influenza A and B, respectively. More details on the transmission model are given in a previous publication [34].

### Vaccination programme

The vaccine efficacy data used for LAIV and TIV were derived from clinical studies and meta-analyses. TIV efficacy among children under 2 years of age, children 2–17 years of age, otherwise healthy adults and ARP was estimated to be 11, 59, 68 and 58 %, respectively [42–45]. We assumed a nearly complete waning for the TIV-induced immunity within 1 year. For LAIV among children we applied an efficacy of 80 % in the first transmission season after vaccination based on the mean value of the estimates of two meta-analyses [45, 46]. A recently published update of a Cochrane meta-analysis confirmed this efficacy data [47]. In accordance with an Asian study [18], we assumed that without revaccination LAIV would still have a remaining efficacy of 56 % in the second season. Details on how we modelled waning immunity are provided in Rose et al. [34]. Adverse events associated with influenza vaccination were only incorporated for children who received LAIV. The probabilities of these adverse events were based on the absolute differences between LAIV and placebo observed in clinical trials within 10 days after the first dose [48, 49]. Current TIV-vaccination rates among children, adults and the elderly in Germany were obtained from a representative population-based cross-sectional survey [50, 51]. During a 14-year run-in phase, the model population was vaccinated with TIV at current age-specific uptake rates. This initial run-in period was followed by a subsequent 10-year intervention phase, where the continuation of TIV use at current vaccination coverage levels was compared to the introduction of an additional routine childhood immunisation programme using LAIV. LAIV coverage was assumed to increase linearly up to 50 % within 3 years, starting with the age-specific baseline coverage at onset of the childhood vaccination programme. In the base-case analysis, annual vaccination takes place in October and November. We assumed a one-dose vaccination scheme. Table 1 provides an overview of the vaccination parameters used in the model.

**Table 1:** Vaccination parameters

Parameter	Value (%)	References
Efficacy in the first transmission season after vaccination		
TIV among children <2 years	11	Vesikari et al. [42]
TIV among children 2–17 years	59	Jefferson et al. [45]
TIV among OHA	68	Monto et al. [44]
TIV among ARP	58	Jefferson et al. [43]
LAIV among children	80	Jefferson et al. [45]; Rhorer et al. [46]
Probability of LAIV-related adverse events		
Runny nose in children 2–6 years	13.5	MedImmune [48]
Runny nose in children 7–17 years	3.9	MedImmune [48]
Headache in children 2–6 years	1.8	MedImmune [48]
Headache in children 7–17 years	6.2	MedImmune [48]
Fever in children 2–6 years	5.5	MedImmune [48]
Fever in children 7–17 years	0.2	MedImmune [48]
Sore throat in children 2–6 years	2.0	MedImmune [48]
Sore throat in children 7–17 years	0.0	MedImmune [48]
Muscle aches in children 2–6 years	2.3	MedImmune [48]
Muscle aches in children 7–17 years	1.9	MedImmune [48]
Vomiting in children 2–6 years	2.5	MedImmune [48]
Vomiting in children 7–17 years	1.5	MedImmune [48]
Yearly baseline vaccination coverage		
Children <1 year	0	Assumption
Children 1–2 years	19.2	Blank et al. [50]
Children 3–6 years	22.4	Blank et al. [50]
Children 7–10 years	23.6	Blank et al. [50]
Children 11–17 years	11	Blank et al. [50]
OHA 18–59 years	14.5	Blank et al. [50]
ARP 18–59 years	29.8	Blank et al. [51]
ARP 60–64 years	33.1	Blank et al. [51]
ARP 65–69 years	47.6	Blank et al. [51]
ARP 70 years and over	53.4	Blank et al. [51]

*OHA* otherwise healthy adults, *ARP* at-risk patients, *TIV* trivalent inactivated influenza vaccine, *LAIV* live attenuated influenza vaccine

### Clinical events and related health care resource utilisation patterns

The fraction of symptomatic influenza cases (66.9 %) was taken from the published literature [40]. Further probabilities of various influenza-associated health outcomes were obtained from previous studies supplemented by expert opinion (see Table 2). The model branches consider health care resource use based on the clinical events that can occur in subjects being infected and having symptomatic influenza with or without further complications. All individuals who develop symptomatic influenza or influenza-associated respiratory complications are given age-specific probabilities for self-medicating with over-the-counter drugs, for consulting a general practitioner, for receiving prescription medication or for being hospitalised. Moreover, hospitalised CAP patients face a low probability of not surviving. All vaccine-related adverse events were assumed to be medically treated at a rate of 30 %, except for runny nose, whose treatment probability was set to 10 %. The probability and average amount of related resource use for each clinical event (e.g. general practitioner visit, prescription medication, hospitalisation) were obtained from published literature or derived by expert consulting using the Delphi technique. The expert panel consisted of six experts specialised in paediatrics, infectious diseases or pulmonology. More details on the Delphi study are given in the paper on the epidemiological model [34]. Key parameters of the influenza outcome subtree are shown in Table 2.

**Table 2:** Probabilities of disease events and resource use

Parameter	Values by age and risk group						References
	CH 1 (%)	CH 2 (%)	CH 3 (%)	CH 4 (%)	OHA (%)	ARP (%)	
Occurrence of disease events							
Developing symptoms, given influenza infection	66.9	66.9	66.9	66.9	66.9	66.9	Carrat et al. [40]
Proportion of patients developing AOM, given symptomatic influenza	39.7	19.6	4.4	4.0	1	1	Heikkinen et al. [9], Meier et al. [52], Sessa et al. [53], expert opinion
Proportion of patients developing CAP, given symptomatic influenza	3.1	2.7	1.1	0.5	0.3	1.3	Heikkinen et al. [9], Meier et al. [52], expert opinion
Not surviving, given CAP	2	1	1	1	4.4	10	Guided by Ewig et al. [54], von Baum et al. [55], expert opinion
Health care consumption							
Proportion of patients requiring physician consultation, given symptomatic influenza	60	40	30	10	20	50	Expert opinion
Proportion of patients requiring antivirals, given physician consultation	1	1	1	1	1	2	Expert opinion
Prescribed antiviral (oseltamivir/zanamivir)	100/0	100/0	100/0	90/10	90/10	90/10	Expert opinion
Proportion of patients (with antiviral therapy) experiencing a beneficial effect	40	40	40	40	40	40	Expert opinion
Prescription of antibiotics, given physician consultation	25	25	25	25	34.5	34.5	Butler et al. [56], expert opinion
Proportion of patients requiring analgesics/antipyretics, given GP consultation	34.3	34.3	34.3	34.3	17.7	36.9	Meier et al. [52]
Proportion of patients requiring antitussives, given GP consultation	30	30	20	20	20	30	Expert opinion
Hospitalisation, given symptomatic influenza	2	1	0.5	0.5	0.5	1	Expert opinion
Self-medicating with OTC medications, given symptomatic influenza	50	50	50	50	50	50	Zok [57], expert opinion
Proportion of patients requiring antibiotic therapy, given AOM	50	40	40	40	50	50	Abbas et al. [58], expert opinion
Proportion of patients requiring analgesics/antipyretics, given AOM	90	90	90	90	90	90	Expert opinion
Proportion of patients requiring further medication (nasal spray), given AOM	90	90	90	90	90	90	Expert opinion
Proportion of patients requiring antibiotic therapy, given CAP	80	70	70	80	80	90	Abbas et al. [58], expert opinion
Proportion of patients requiring analgesics/antipyretics, given CAP	80	80	80	80	80	80	Expert opinion
Proportion of patients requiring further medication (antitussives), given CAP	60	60	60	60	60	60	Expert opinion
Proportion of patients requiring outpatient chest x-ray, given CAP	81	81	50	50	50	80	Weigl et al. [59], expert opinion
Hospitalisation, given CAP	65	50	30	30	64	64	von Baum et al. [55], expert opinion
ICU, given CAP hospitalisation	10	5	2	2	2	9	Ewig et al. [60], expert opinion

*CH 1* children <2 years, *CH 2* children 2–6 years, *CH 3* children 7–12 years, *CH 4* adolescents 13–17 years, *OHA* otherwise healthy adults, *ARP* at-risk patients, *AOM* acute otitis media, *CAP* community-acquired pneumonia, *OTC* over-the-counter, *GP* general practitioner, *ICU* intensive care unit

### Cost data

As stated in a previous section, model analyses were performed from three perspectives. The narrow third-party payer perspective comprises only reimbursed direct medical costs which include vaccination costs as well as treatment costs for influenza and its sequelae. In the broad third-party payer perspective, transfer payments associated with parental absence from work due to illness of children up to 12 years were additionally included. These transfer payments comprise the reimbursement of 70 % of the work-loss costs of employed parents (Kinderpflegekrankengeld, i.e. child care sickness benefits) for up to 10 days per child per year by German statutory sickness funds. In the societal perspective, patient co-payments for physician office visits, prescription medication and inpatient services as well as costs of self-medicating with over-the-counter drugs were included alongside with reimbursed direct medical costs. Furthermore, indirect costs associated with production losses due to sick leave and premature death were considered in the societal perspective.

Unit costs for health care utilisation were drawn from German sources only. Drug costs were derived from a German pharmaceutical database called Lauer-Taxe [61] using January 2012 information and considering current manufacturer rebates as well as pharmacy discounts. Antibiotic therapy was amoxicillin, or in the case of CAP treatment in ARP, amoxicillin and clavulanic acid combination. Paracetamol (acetaminophen) was used as standard analgesic and antipyretic therapy. Antitussive therapy consisted of ambroxol and noscapine. We assumed that typical self-medication includes use of pain relievers, nasal spray and cough medicine. The price per dose of TIV was estimated at €10.64. Vaccine acquisition cost per dose of LAIV was assumed to be €20.20. This information was provided by the manufacturer of LAIV (AstraZeneca/MedImmune). The vaccine administration fee of €6.65 was based on the mean influenza immunisation fee in Germany. This estimate was the result of a survey of the 17 Associations of Statutory Health Insurance Physicians (ASHIPs).

Unit costs for treatment-related physician visits and outpatient diagnostic procedures were based on official tariffs derived from the German physician fee scale called EBM (Einheitlicher Bewertungsmaßstab) [62] using a point value of €0.035048 [63]. Hospitalisation costs were taken from the German DRG (diagnosis-related group) catalogue [64] considering a base rate of €2,935.78. We applied the group codes E77G and D62Z for inpatient treatment of influenza. Hospitalisation of CAP patients was split up into inpatient stays with and without an intensive care unit (ICU) admission. CAP-associated hospitalisation in a general ward was grouped into DRG E77G. Hospitalisation of patients with CAP requiring intensive care was classified as DRGs E40C and A13G.

Indirect costs were calculated according to the friction cost approach [65] using a friction period of 56 days. This figure corresponds to the average vacancy period in Germany in 2010 [66]. The average number of work days lost attributable to influenza, AOM and CAP were obtained from an administrative database of a German sickness fund using 2008 data [67], weighted by current age-specific employment rates. Estimates of parental absence from work to care for a sick child were taken from a Finnish study [9]. The average cost per work day lost was calculated using national statistics on income and employment figures and updated to 2012 values applying the nominal wage growth rate. We assumed the nominal wage growth rate for 2012 to be the same as for 2011 (3.3 %). On that basis, mean daily income per employed person was estimated to be €90.84.

All costs are reported in euro (€) at 2012 price level. All future costs and benefits were discounted at 3 % according to German guidelines on economic evaluation in health care [68]. An overview of the direct cost parameters used in the model is given in Table 3. Indirect cost inputs are shown in Table 4.

**Table 3:** Direct unit costs

Parameter	Costs (€) by age and risk group (TPP; PCP)							References
	CH 1	CH 2	CH 3	CH 4	OHA	ARP <60	ARP 60+	
Vaccination (per dose)								
TIV	10.64; N/A	10.64; N/A	10.64; N/A	10.64; N/A	10.64; N/A	10.64; N/A	10.64; N/A	Lauer-Taxe [61]
LAIV	N/A; N/A	20.02; N/A	20.02; N/A	20.02; N/A	N/A; N/A	N/A; N/A	N/A; N/A	Lauer-Taxe [61]
Vaccine administration fee	6.65; N/A	6.65; N/A	6.65; N/A	6.65; N/A	6.65; N/A	6.65; N/A	6.65; N/A	ASHIPs
Treatment of adverse events^a^								
GP consultation	N/A; N/A	41.71; N/A	30.84; N/A	30.84; N/A	N/A; N/A	N/A; N/A	N/A; N/A	EBM catalogue [62]
Runny nose	N/A; N/A	1.76; N/A	2.16; N/A	N/A; 2.38	N/A; N/A	N/A; N/A	N/A; N/A	Lauer-Taxe [61]
Headache	N/A; N/A	2.83; N/A	1.27; N/A	N/A; 1.34	N/A; N/A	N/A; N/A	N/A; N/A	Lauer-Taxe [61]
Fever	N/A; N/A	2.83; N/A	1.27; N/A	N/A; 1.34	N/A; N/A	N/A; N/A	N/A; N/A	Lauer-Taxe [61]
Sore throat	N/A; N/A	2.83; N/A	6.33; N/A	N/A; 7.27	N/A; N/A	N/A; N/A	N/A; N/A	Lauer-Taxe [61]
Muscle aches	N/A; N/A	2.83; N/A	1.27; N/A	N/A; 1.34	N/A; N/A	N/A; N/A	N/A; N/A	Lauer-Taxe [61]
Vomiting	N/A; N/A	3.06; N/A	3.29; N/A	N/A; 3.63	N/A; N/A	N/A; N/A	N/A; N/A	Lauer-Taxe [61]
Treatment of influenza^b^								
GP consultation	41.71; N/A	41.71; N/A	30.84; N/A	30.84; N/A	20.84; 10.00	20.84; 10.00	25.75; 10.00	EBM catalogue [62]
Oseltamivir	20.05; N/A	31.68; N/A	31.68; N/A	31.68; N/A	26.86; 5.00	26.86; 5.00	26.86; 5.00	Lauer-Taxe [61]
Zanamivir	N/A; N/A	N/A; N/A	N/A; N/A	28.59; N/A	23.59; 5.00	23.59; 5.00	23.59; 5.00	Lauer-Taxe [61]
Antibiotics	10.02; N/A	10.02; N/A	11.33; N/A	11.33; N/A	6.33; 5.00	6.33; 5.00	6.33; 5.00	Lauer-Taxe [61]
Analgesics and antipyretics	1.09; N/A	2.83; N/A	1.27; N/A	N/A; 1.34	N/A; 1.34	N/A; 1.34	N/A; 1.34	Lauer-Taxe [61]
Antitussives	7.66; N/A	7.66; N/A	7.66; N/A	5.85; 2.00	3.35; 4.50	3.35; 4.50	3.35; 4.50	Lauer-Taxe [61]
Self-medication	N/A; 6.32	N/A; 9.05	N/A; 7.31	N/A; 7.31	N/A; 7.31	N/A; 7.31	N/A; 7.31	Lauer-Taxe [61]
Hospitalisation	2,012.10; N/A	1,986.97; N/A	1,986.97; N/A	1,986.97; N/A	1,926.97; 60.00	1,926.97; 60.00	1,926.97; 60.00	DRG catalogue [64]
Treatment of AOM								
Antibiotics	10.02; N/A	10.02; N/A	11.33; N/A	11.33; N/A	6.33; 5.00	6.33; 5.00	6.33; 5.00	Lauer-Taxe [61]
Analgesics and antipyretics	1.09; N/A	2.83; N/A	1.27; N/A	N/A; 1.34	N/A; 1.34	N/A; 1.34	N/A; 1.34	Lauer-Taxe [61]
Nasal spray	1.09; N/A	1.76; N/A	2.16; N/A	N/A; 2.38	N/A; 2.38	N/A; 2.38	N/A; 2.38	Lauer-Taxe [61]
Treatment of CAP								
Antibiotics	11.93; N/A	12.00; N/A	12.00; N/A	12.00; N/A	8.09; 5.00	42.40; 5.42	42.40; 5.42	Lauer-Taxe [61]
Analgesics and antipyretics	1.09; N/A	2.83; N/A	1.27; N/A	N/A; 1.34	N/A; 1.34	N/A; 1.34	N/A; 1.34	Lauer-Taxe [61]
Outpatient chest x-ray	20.33; N/A	20.33; N/A	19.45; N/A	19.45; N/A	19.45; N/A	19.45; N/A	20.33; N/A	EBM catalogue [62]
Antitussives	7.66; N/A	7.66; N/A	7.66; N/A	5.85; 2.00	3.35; 4.50	3.35; 4.50	3.35; 4.50	Lauer-Taxe [61]
Hospitalisation, general ward	2,174.84; N/A	2,174.84; N/A	2,174.84; N/A	2,174.84; N/A	2,108.84; 66.00	2,108.84; 66.00	2,108.84; 66.00	DRG catalogue [64]
Hospitalisation, ICU	7,015.14; N/A	7,015.14; N/A	7,015.14; N/A	7,015.14; N/A	6,916.34; 98.80	6,916.34; 98.80	6,916.34; 98.80	DRG catalogue [64]

*TPP* third-party payer, *PCP* patient co-payments, *CH 1* children <2 years, *CH 2* children 2–6 years, *CH 3* children 7–12 years, *CH 4* adolescents 13–17 years, *OHA* otherwise healthy adults, *ARP* <*60* at-risk patients under the age of 60 years, *ARP 60*+ at-risk patients aged 60 years and over, *TIV* trivalent inactivated influenza vaccine, *LAIV* live attenuated influenza vaccine, *AOM* acute otitis media, *CAP* community-acquired pneumonia, *GP* general practitioner, *ICU* intensive care unit, *ASHIPs* Associations of Statutory Health Insurance Physicians, *N/A* not applicable

^a^LAIV-associated adverse events

^b^Symptomatic cases of influenza

**Table 4:** Indirect costs

Parameter	Indirect costs (€) by age (in years)										References (work days lost)
	15–19	20–24	25–29	30–34	35–39	40–44	45–49	50–54	55–59	60–64	
(Additional) work days lost											
Symptomatic influenza^a^	160.12	362.03	426.42	452.86	468.59	480.34	475.21	453.88	400.16	219.95	AOK Bundesverband [67]
AOM, given symptomatic influenza^b^	12.71	28.74	33.85	35.94	37.19	38.13	37.71	36.02	31.76	17.46	
CAP, given symptomatic influenza^c^	256.70	580.40	683.62	726.02	751.23	770.06	761.84	727.65	641.52	352.61	
CAP death, given CAP^d^	1,006.44	2,275.61	2,680.35	2,846.57	2,945.43	3,019.27	2,986.99	2,852.96	2,515.28	1,382.51	Bundesagentur für Arbeit [66]

Parameter	Indirect costs (€) by age group				References (work days lost)
	CH 1	CH 2	CH 3	CH 4	
Parental work days lost due to child’s symptomatic influenza^e^	177.91	132.20	50.17	0.00	Calculated using data from Heikkinen et al. [9]

*AOM* acute otitis media, *CAP* community acquired pneumonia, *CH 1* children <2 years, *CH 2* children 2–6 years, *CH 3* children 7–12 years, *CH 4* adolescents 13–17 years

^a^6.3 days

^b^0.5 days (6.8 − 6.3 = 0.5)

^c^10.1 days (16.4 − 6.3 = 10.1)

^d^39.6 days (56 − 16.4 = 39.6)

^e^2 days (CH 1); 1.5 days (CH 2); 0.6 days (CH 3); 0 days (CH 4)

### Health-related quality of life

Influenza-associated symptoms and complications cause specific reductions in quality of life. Utility values for influenza and influenza-related complications were based on published literature and previous modelling studies. As a result of the lack of specific quality of life estimates for a German population, we used international data. The quality of life weight for each day of uncomplicated influenza or influenza-related AOM was 0.56 [69, 70]. In the study by Mauskopf et al. [69] the utility weight for influenza illness was obtained using the quality of well-being scale which combines a description of the functional status with a problem symptom complex. The utility value for each day with CAP was assumed to be 0.52 [70]. This quality of life weight was derived using data on health states measured by activity limitation and perceived health. Average durations of disease states ranging from 4 to 18 days were based on expert opinion. Because the established side effects of LAIV are of mild severity, QALY losses due to the occurrence of adverse events were not considered in this modelling approach. We assumed a utility of 1 without symptomatic influenza or associated diseases. Furthermore premature death due to CAP resulted in a QALY loss based on the remaining life expectancy.

### Time horizon

After a run-in phase of 14 years, using merely current age-specific TIV-coverage rates, the model followed the entire German population over additional 10 years in order to estimate the effects of a supplementary general childhood influenza vaccination in Germany. The analytic horizon of 10 years was chosen to capture introductory effects of the new vaccination policy and to account for seasonal variations in influenza epidemiology.

### Sensitivity analyses

We performed a series of sensitivity analyses to evaluate the robustness of the model. Several deterministic one-way sensitivity analyses were carried out to test how separate changes in key variables or assumptions affected the results. Ranges (given in brackets) are based on published literature or expert opinion. The varied parameters include natural history parameters, vaccination parameters and economic parameters:

- Basic reproduction number (1.3/2.1)
- Duration of viral shedding (3 days/7 days)
- Duration of naturally acquired immunity (±4 years)
- LAIV efficacy (±10 %)
- Halving of childhood vaccination coverage at baseline
- LAIV coverage among children (30 %/70 %)
- Target age range for LAIV (2–6 instead of 2–17 years)
- Use of TIV instead of LAIV
- Halving of the proportion of children seeking outpatient treatment
- Disease events including symptomatic cases, AOM, CAP and death of pneumonia (±20 %)
- Vaccine price of LAIV (±20 %)
- Direct treatment costs (±20 %)
- Transfer payments (±20 %)
- Discount rate (0 %/5 %)
- Adjusting the health state utilities for age-specific baseline values from the general population instead of assuming a baseline of perfect health [71].

A two-way sensitivity analysis considered different estimates of two parameters: the coverage rate and the maximum target age of the routine childhood immunisation programme.

Furthermore, a probabilistic sensitivity analysis was conducted to explore the overall uncertainty by varying all major model parameters simultaneously using a random number generator and appropriate distributions. The lognormal distribution was assigned to cost parameters and some transmission characteristics of influenza (basic reproduction number, duration of infectious period and duration of natural immunity), whereas the beta distribution was applied to probabilities, utilities and efficacy estimates.

### Model validation

Validation analysis was performed by comparing the number of outpatient visits predicted by our model using current vaccination uptake rates with the excess consultations attributed to influenza estimated on the basis of German surveillance data [21]. Comparing the average number of physician consultations per year simulated by our model with the observed age-specific excess consultations associated with influenza from the 2001–2002 to the 2010–2011 season showed that simulated outpatient visits lay mostly below the observed rates of excess consultations. Furthermore, the simulated number of CAP-related deaths per year was rather low when compared to the average influenza-associated excess deaths reported for Germany [72, 73]. Hence, our model tends to underestimate clinical outcomes on seasonal influenza epidemics, which can be considered a conservative approach.

## Results

### Epidemiological impact

The number of prevented cases of several clinical outcomes was used to measure the population-level effects. Under base-case assumptions, annual routine vaccination of children with LAIV would prevent, across all ages, an estimated 16 million cases of symptomatic influenza, resulting in a reduction of 600,968 cases of AOM and 128,861 cases of CAP over 10 years in Germany if left undiscounted. Furthermore, an average of 506 pneumonia-related deaths would be averted per year. Most of the avoided deaths would be prevented in adults and the elderly. Because of the decrease in the burden of disease, an average of 168,239 prescriptions of antibiotics and 16,712 hospitalisations could be prevented annually. Owing to indirect protection provided by the childhood vaccination programme, about 60 % of the prevented hospitalisations would appear in adults and the elderly. Table 5 presents the undiscounted epidemiological results of the base-case analysis in terms of total cases across all age groups.

**Table 5:** Epidemiological results of the base-case analysis

Undiscounted 10-year outcomes (overall cases across all age groups)	Current policy	Current policy + LAIV-based routine childhood vaccination (2–17 years)	Difference (total cases prevented)	Distribution of avoided cases by age group	
				Under 18 years (%)	18 years and over (%)
Infections	58,863,475	34,958,394	23,905,081	38	62
Symptomatic cases	39,379,665	23,387,166	15,992,499	38	62
Cases of AOM	1,145,311	544,343	600,968	83	17
Cases of CAP	282,447	153,586	128,861	57	43
Deaths	13,960	8,902	5,058	16	84
Prescribed antibiotics	4,172,573	2,490,181	1,682,392	38	62
Hospitalisations	406,297	239,178	167,119	42	58

*AOM* acute otitis media, *CAP* community-acquired pneumonia, *LAIV* live attenuated influenza vaccine

### Cost-effectiveness

A summary of the underlying cost analysis is given in Table 6. The discounted cost-effectiveness results of the base-case analysis are shown in Table 7. From a narrow third-party payer perspective, the discounted incremental cost-effectiveness ratio of a seasonal influenza immunisation policy including routine childhood vaccination using LAIV was €2,265 per QALY gained, when compared to the current strategy of vaccinating primarily risk groups with TIV. The corresponding return rate per euro invested was 0.52 from that perspective. From a broad third-party payer perspective, which takes into account child care sickness benefits, the incremental cost-effectiveness ratio was €1,228 per QALY gained and the return rate per euro invested increased to 0.74. From the societal perspective, the inclusion of patient co-payments and indirect costs in terms of production losses resulted in discounted 10-year cost savings of €3.4 billion. According to this overall cost-offset, the return rate per euro invested was 5.07 when taking a societal perspective. In other words, the introduction of routine childhood influenza vaccination would save €5.07 for each euro invested in the childhood immunisation programme.

**Table 6:** Summary of the cost analysis using base-case estimates

Cost category	Discounted 10-year costs (€)		
	CP	CP + RCHV	Difference
Direct medical costs of vaccination against influenza (TPP)			
TIV	1,872,816,214.16	1,701,799,776.42	−171,016,437.72
Administration of TIV	1,170,510,133.83	1,063,624,860.26	−106,885,273.57
LAIV	0.00	791,516,964.16	791,516,964.16
Administration of LAIV	0.00	262,916,474.11	262,916,474.11
Treatment of LAIV-associated adverse events	0.00	57,983,157.76	57,983,157.76
Direct medical costs of treating influenza-related diseases (TPP)			
Outpatient medical treatment	239,528,399.93	137,833,556.65	−101,694,843.28
Outpatient pharmaceutical treatment	47,278,534.57	26,436,026.60	−20,842,507.97
Inpatient treatment	759,862,529.73	446,500,962.87	−313,361,566.86
Transfers and indirect costs			
Transfers (Kinderpflegekrankengeld)	302,065,027.59	119,571,107.09	−182,493,920.50
Indirect costs in terms of production losses	10,708,705,718.42	6,997,244,130.30	−3,711,461,588.12
Total costs			
Narrow TPP perspective	4,089,995,812.19	4,448,611,778.81	398,615,966.62
Broad TPP perspective	4,392,060,839.78	4,608,182,885.90	216,122,046.12
Societal perspective (including co-payments and indirect costs)	15,042,784,059.11	11,639,184,713.27	3,403,599,345.84

*CP* current policy, *RCHV* LAIV-based routine childhood vaccination (2–17 years), *TPP* third-party payer, *TIV* trivalent inactivated influenza vaccine, *LAIV* live attenuated influenza vaccine

**Table 7:** Economic results of the base-case analysis

Discounted 10-year outcomes	Narrow TPP perspective		Broad TPP perspective		Societal perspective	
	CP	CP + RCHV	CP	CP + RCHV	CP	CP + RCHV
Direct costs (€)	4,089,995,812	4,488,611,779	4,089,995,812	4,488,611,779	4,334,078,341	4,641,940,583
Transfers (€)	N/A	N/A	302,065,028	119,571,107	N/A	N/A
Indirect costs (€)	N/A	N/A	N/A	N/A	10,708,705,718	6,997,244,130
Total costs (€)	4,089,995,812	4,488,611,779	4,392,060,840	4,608,182,886	15,042,784,059	11,639,184,713
Lost QALYs	449,443	273,483	449,443	273,483	449,443	273,483
ICER (€/QALY)		2,265		1,228		Strategy is dominant
Return rate		0.52		0.74		5.07

*CP* current policy, *RCHV* LAIV-based routine childhood vaccination (2–17 years), *TPP* third-party payer, *QALY* quality-adjusted life year, *ICER* incremental cost-effectiveness ratio, *N/A* not applicable

### Sensitivity analyses

**Fig. 2:**
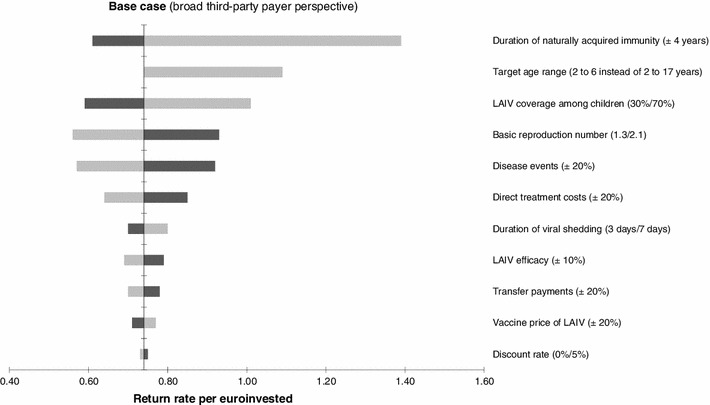
Results of one-way sensitivity analyses on key model parameters (the *dark bars* represent the upper limits whereas the *light bars* indicate the lower limits). *LAIV* live attenuated influenza vaccine

We performed a range of one-way sensitivity analyses to explore the effect of varying key input parameters on economic results. Taking a broad third-party payer perspective into account, the results were sensitive to changes in the duration of immunity induced by natural influenza infection, the influenza vaccination coverage rate in children and the target age range of the childhood immunisation programme (parameters are listed in the order of strength). For instance, reducing the maximum age limit of target age range from 2–17 to children 2–6 years of age (while keeping the base-case coverage rate for LAIV at 50 %) increased the return rate to 1.09/€ invested. Cost-saving results were achieved up to a recommended vaccination age of 7 years when adopting a broad third-party payer perspective. Sensitivity analyses on the duration of viral shedding, transfer payments and vaccine price for LAIV showed moderate to marginal impact on the cost-effectiveness results. Halving of the childhood vaccination coverage at baseline led to slightly increased cost-effectiveness ratios (and thus decreased return rates) at an overall increase in cases prevented. Halving of the proportion of children seeking outpatient treatment also led to increased cost-effectiveness ratios (such as €1,377 per QALY gained from a broad third-party payer perspective). A similar effect was observed when adjusting the health state utilities for age-specific baseline values (€1,425 per QALY gained from a broad third-party payer perspective). Compared to the base-case return rate of 0.74, implementing the routine childhood immunisation programme using TIV instead of LAIV was associated with a lowered return rate per euro invested (0.63). In this scenario, the number of prevented symptomatic influenza cases decreased (from 1.6 million/year) to an estimated 600,000/year and the reduction in hospitalisations decreased from 16,712/year in the base-case to 6.444/year. Figure 2 summarises the economic results of various one-way sensitivity analyses using a tornado chart. Table 8 displays the prevented cases for different outcome measures due to childhood vaccination against influenza using LAIV or TIV at different immunisation uptake levels. Furthermore, we conducted a two-way sensitivity analysis, where the target age range of the childhood immunisation programme and the vaccine uptake of LAIV were varied simultaneously. The results of this analysis are presented in Table 9.

**Table 8:** Annual averted disease burden across all age groups by vaccine type and coverage rate

Outcome measure	Average avoided cases per year by vaccine type and coverage rate among children and adolescents 2–17 years of age (uptake is indicated in brackets)					
	LAIV (30 %)	LAIV (50 %)	LAIV (70 %)	TIV (30 %)	TIV (50 %)	TIV (70 %)
Influenza infections	1,652,683	2,390,508	2,852,758	375,220	900,924	1,380,496
Symptomatic influenza cases	1,105,645	1,599,250	1,908,495	251,022	602,718	923,552
Cases of AOM	42,707	60,097	70,226	10,399	25,897	39,049
Cases of CAP	9,050	12,886	15,199	2,118	5,244	7,965
Prescribed antibiotics	115,984	168,239	200,972	25,973	64,131	98,466
Hospitalisations	11,543	16,712	19,933	2,616	6,444	9,875
Deaths	343	506	611	75	184	286

*AOM* acute otitis media, *CAP* community-acquired pneumonia, *LAIV* live attenuated influenza vaccine, *TIV* trivalent inactivated influenza vaccine

**Table 9:** Results of a two-way sensitivity analysis varying the target age range of the routine childhood vaccination programme and the vaccine uptake of LAIV adopting a broad third-party payer perspective

LAIV coverage rate (%)	Return rates for different target age ranges (in years) of the routine childhood vaccination programme											
	2–6	2–7	2–8	2–9	2–10	2–11	2–12	2–13	2–14	2–15	2–16	2–17
30	1.33	1.31	1.28	1.26	1.25	1.20	1.15	1.11	1.08	1.05	1.03	1.01
50	1.09	1.03	0.98	0.94	0.92	0.88	0.84	0.81	0.79	0.77	0.76	0.74^a^
70	0.92	0.85	0.80	0.76	0.74	0.71	0.67	0.65	0.63	0.62	0.60	0.59

*LAIV* live attenuated influenza vaccine

^a^Base case

**Fig. 3:**
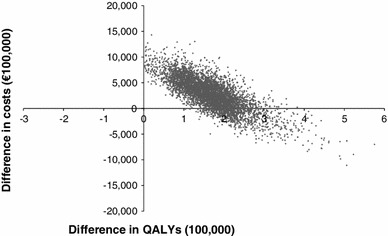
Results of the probabilistic sensitivity analysis (50 % LAIV coverage; broad third-party payer perspective). *QALY* quality-adjusted life year

**Fig. 4:**
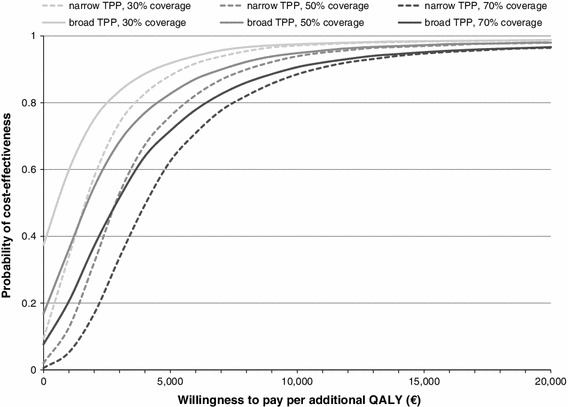
Cost-effectiveness acceptability curves for different LAIV coverage rates. *TPP* third-party payer, *QALY* quality-adjusted life year

A probabilistic sensitivity analysis was performed using Monte Carlo simulation and results were based on 5,000 simulation runs. Figure 3 illustrates the uncertainty surrounding the cost-effectiveness estimate assuming 50 % LAIV coverage of children 2–17 years of age and adopting a broad third-party payer perspective. The scatter plot shows that routine childhood vaccination with LAIV was cost saving in 17 % of the simulation runs. Figure 4 presents cost-effectiveness acceptability curves for different LAIV coverage rates adopting either a narrow or a broad third-party payer perspective. As the graph shows, the introduction of an influenza immunisation policy including routine childhood vaccination with LAIV and considering an LAIV uptake of 30 % had a 37 % probability of being cost saving from a broad third-party payer perspective. Increasing the LAIV coverage level led to lower probabilities of being cost saving. However, all scenarios were associated with a greater than 95 % probability of being cost-effective at a willingness to pay threshold of €20,000 per QALY.

## Discussion

This is the first paper to assess the potential cost-effectiveness of a childhood seasonal influenza immunisation with LAIV in Germany. In this study, a dynamic transmission model was used to estimate 10-year outcomes in terms of costs and various disease consequences of a routine childhood influenza vaccination programme in addition to the current practice of focussing on people with chronic conditions and the elderly.

On the basis of our model projections, the introduction of a routine childhood influenza vaccination programme assuming 50 % coverage and use of LAIV could lead to a significant decrease in influenza infections resulting in decreased morbidity and mortality across all age groups, reduced hospitalisation rates and antibiotic use as well as less sickness-related absence from work. Besides direct effects in children, significant parts of the overall benefits (up to 84 %) would be caused by indirect effects of the childhood vaccination programme in people aged 18 and older.

In Germany, as in most countries, decision makers (Federal Joint Committee) have not defined thresholds for cost-effectiveness so far. Nonetheless, considering the commonly accepted threshold of €50,000 per QALY, the introduction of a routine childhood vaccination programme appears to be highly cost-effective from a narrow third-party payer perspective (accounting for reimbursed direct health care costs only) and from a broad third-party payer perspective (including transfer payments for parental absence from work). Moreover, our results indicate that the introduction of a routine childhood influenza vaccination programme using LAIV could even lead to overall cost savings. Meaningful cost-offsets were identified when taking a societal perspective. Hence, cost savings were mainly driven by the inclusion of indirect costs. Results of univariate sensitivity analyses suggest that cost savings could also be realised by decreasing the number of children who receive vaccination, particularly by limiting the target age group to children under 8 years of age when taking a broad third-party payer perspective. In addition, our probabilistic sensitivity analysis revealed that, depending on the uptake, routine influenza vaccination of children was cost saving in up to 37 % of the simulations performed from the broad third-party payer perspective. Probabilistic sensitivity analysis also showed that routine influenza vaccination of children had a very high probability of being cost-effective at a willingness to pay threshold of €20,000 per QALY.

A number of previous studies carried out in different countries have assessed the cost-effectiveness of influenza vaccination in children (see, for example, overviews by Nichol [74] and Savidan et al. [75]). Compared to the present analysis, some of these studies provided similar findings.

In a modelling study from Argentina [76], influenza vaccination of high-risk children aged 6 months to 15 years old was estimated to be cost saving from a societal perspective. A US-based study [77] demonstrated that the probability of generating cost savings was highest when vaccinating high-risk children. Furthermore, indirect costs were identified as the main drivers of cost savings. Another US-based study [78] assessing the economic impact of influenza vaccination in children found that immunisation of healthy school-aged children was cost saving, primarily owing to avoided indirect costs. An Italian study [79] also reported cost-saving results from a societal perspective when comparing a universal vaccination programme with an adjuvanted influenza vaccine in children aged 6–60 months to current immunisation practice. A study from Finland [80] evaluated the cost-effectiveness of influenza vaccination of healthy children 6 months to 13 years of age from a health care provider and a societal perspective. The authors concluded that a general vaccination of healthy children would be cost saving from both perspectives considered. In contrast, a Canadian study [81] analysing the cost-effectiveness of annual influenza vaccination for healthy infants and toddlers aged 6–23 months concluded that influenza immunisation was not cost saving for this age group from both a third-party payer and a societal perspective.

To date, only few economic model analyses explicitly addressed the use of LAIV. One study from the USA [82] evaluated the cost-effectiveness of LAIV relative to TIV in children aged 24–59 months assuming a societal perspective. The authors found that, compared to TIV, vaccinating children with LAIV was associated with cost savings due to higher efficacy of LAIV. Another US-based study [83], assessing the economic impact of childhood influenza vaccination relative to no vaccination, projected cost-effectiveness ratios of $15,000 per QALY for LAIV and $18,000 per QALY for TIV when vaccinating non-high-risk children aged 2 years; but compared to previously mentioned studies, the authors did not consider indirect costs due to parental absence from work associated with influenza. A study which adopted a societal perspective and therefore included parental work-loss costs [84] found that the use of LAIV resulted in net cost savings when the cost per dose was at or below $36 assuming no parental absence from work to obtain childhood influenza vaccination.

However, none of these studies have assessed the full economic impact of routine childhood vaccination against influenza by use of a dynamic transmission model. As a consequence, the cost-effectiveness of influenza vaccination in children has been underestimated. So far, we are aware of only two studies [85, 86] that applied a transmission model and reported economic effects on vaccinating children against influenza. Unfortunately, the US-based study by Weycker et al. [86] only specified cost-offsets due to prevented illnesses but did not incorporate vaccination costs. In contrast, Giglio et al. [85] reported results of a full economic evaluation of a paediatric influenza vaccination programme in Argentina, taking into account direct and indirect benefits of vaccinating children from 6 months up to 5 years of age. The results of this recently published study indicate that an influenza vaccination programme targeting preschool-aged children is cost-effective from a direct cost perspective. In comparison to our model, the Argentinian simulation study is based on a different modelling approach which deals with interactions between individuals living in the same or different households, neighbourhoods and communities and belonging to various age-related activity groups.

The major strength of our analysis is that, unlike most previously published economic studies, we used a dynamic modelling approach to capture not only direct effects but also population-wide benefits of a universal childhood influenza immunisation programme. On the other hand, as with any modelling study, there are potential limitations that should be considered when interpreting the findings. First, our model assumed a one-dose vaccination scheme for both vaccines, even regarding previously unvaccinated individuals. The administration of two initial doses to previously unvaccinated children would increase vaccination costs and could alter the cost-effectiveness results. However, the rate of receipt of two doses is low [87, 88] and LAIV has provided high efficacy following a single dose in previously unvaccinated young children [89]. Second, the data on efficacy of TIV in children we used in our model is based on the conventional, non-adjuvanted formulation of the inactivated vaccine. An adjuvanted version of TIV demonstrated high vaccine efficacy in children and was found to be more efficacious compared to the non-adjuvanted vaccine [42]. However, the manufacturer of the adjuvanted vaccine withdrew its application for paediatric-use marketing authorisation in Europe. Third, as a result of a lack of detailed data, assumptions about the immunity to natural influenza infection rely solely on a previous modelling study. Furthermore, missing age-specific data on natural history estimates and resource utilisation patterns were derived from expert opinion. Fourth, simulated influenza-related complications were limited to AOM and CAP. However, influenza can cause other severe as well as costly illnesses and even life-threatening complications. As a consequence, our model may underestimate the real impact of a routine childhood vaccination. Fifth, comorbidities were not considered when calculating hospitalisation costs, thus resulting in an underestimation of these cost components. Finally, we could not conduct extensive validation analyses as a result of a lack of data on different influenza-associated events in Germany. However, the results of validation analysis using excess consultations suggest that the model projects quite realistic but conservative scenarios.

## Conclusions

In deciding whether to reimburse new health care interventions, decision makers increasingly consider both the health effects and the potential economic implications of the different programmes under consideration. Thus, we conducted a cost-effectiveness analysis of a general influenza vaccination programme in children and adolescents using LAIV in Germany.

Taking cost-effectiveness ratios of €2,265 and €1,228 per QALY gained into account, annual routine vaccination of children 2–17 years of age with LAIV appears a highly cost-effective option from a narrow and a broad third-party payer perspective, respectively. When adopting a societal perspective, routine vaccination of children and adolescents against seasonal influenza with LAIV appears a cost-saving strategy.

Compared to the current vaccination policy, the introduction of a universal childhood vaccination programme using LAIV can substantially increase benefits and reduce the influenza-associated burden of disease in Germany. Furthermore, our model results suggest that routine influenza vaccination targeting children and adolescents offers not only advantages for the target group, but provides significant health benefits to the whole population.

In summary, adopting universal influenza immunisation of healthy children and adolescents provides good value for money for the German statutory health insurance system. On the basis of the epidemiological and health economic simulation results, the implementation of annual routine influenza vaccination of children 2–17 years of age should be taken into consideration. Taking into account the efficacy profile, the convenient and painless route of administration as well as the results of our model analysis, live virus vaccines might be an important part of a general influenza vaccination programme for children from the age of two upwards.
